# Post-exposure persistence of nitric oxide upregulation in skin cells irradiated by UV-A

**DOI:** 10.1038/s41598-022-13399-4

**Published:** 2022-06-08

**Authors:** Gareth Hazell, Marina Khazova, Howard Cohen, Sarah Felton, Ken Raj

**Affiliations:** 1 UK Health Security Agency, Chilton, Didcot, OX11 0RQ UK; 2 Elizabeth House, 515 Limpsfield Road, Warlingham, CR6 9LF Surrey UK; 3 Oxford University Hospitals NHS Foundation Trust, Old Road, Oxford, OX3 7LJ UK

**Keywords:** Cell biology, Molecular biology, Natural hazards

## Abstract

Evidence suggests that exposure to UV-A radiation can liberate nitric oxide from skin cells eliciting vasodilation in-vivo. However, the duration of nitric oxide release in skin cells after UV exposure is not well studied, with emphasis on UV-B mediated iNOS upregulation. The current study demonstrated persistence of nitric oxide release in a dark reaction after moderate UV-A exposure, peaking around 48 h post exposure; this effect was shown in keratinocytes, fibroblasts and endothelial cells from neonatal donors and keratinocytes from aged donors and confirmed the hypothesis that UV-A exposure appeared to upregulate cNOS alongside iNOS. Release of nitric oxide in the skin cells induced by a moderate exposure to UV-A in sunlight may be especially beneficial for some demographic groups such as the elderly, hypertensive patients or those with impaired nitric oxide function, not only during exposure but many hours and days after that.

## Introduction

For a simple molecule, nitric oxide (NO) has a somewhat chequered history^1,2^. When first discovered, due to its incredibly short half-life and non-protein nature, it was simply seen as an ‘end-product’ of metabolic processes^3,4^. Opinion changed some 25 years ago when a cardioprotective signalling molecule termed ‘endothelium derived relaxation factor’ (EDRF) was found to be NO^5^. Indeed, NO has since been recognised as the most potent vasodilatory signalling molecule, playing key roles in cardio-protection^6^, causing alleviation of conditions including high blood pressure, heart disease and stroke^7–10^. The ascent of NO’s importance is even more surprising given its classification as a common environmental toxin found at high quantities in car exhaust and cigarette smoke^5^.

Generation of NO in vivo has been commonly associated with enzymatic activity through nitric oxide synthase (NOS)^11,12^. It is now widely acknowledged the enzyme’s ability to supply the body with nitric oxide wanes as we age^13–16^. However, like many complex systems, the body has adapted to this ageing phenomenon, with research suggesting that the loss of NOS in vivo may be compensated via increased breakdown of metabolites such as nitrite, and nitroso-thiols into NO^17–19^. Generation of NO through this metabolite-driven pathway within the skin involves sunlight, with ultraviolet A (UV-A) radiation eliciting breakdown of these compounds held at high concentrations in keratinocytes which constitute over 90% of the skin’s epidermis^20,21^. Support for this effect of sunlight is found in epidemiological studies showing that countries lying closer to the equator, with highest sunlight hours in winter months, present lower cardiovascular incidences than those nearer the Poles, with shortest sunlight hours^22,23^. This has instigated research into the potential effects of sunlight in the restoration of nitric oxide level in compromised individuals^24–26^.

We have previously confirmed the effectiveness of UV-A in generating NO in skin cells^25^. Further studies reported here evaluate the dynamics of nitric oxide production over post-exposure time, and have observed that many hours after exposure to UV-A at a dose that is equivalent to 30 min on a summer’s day in the UK, skin cells continue to produce nitric oxide in the absence of light. We also report here the possible mechanism of this prolonged NO release, and the effectiveness of longer UV-A wavelengths in inducing these effects in skin cells.

## Materials and methods

### Cell culture

Cell culture and skin sample extraction was carried out as previously described by Holliman et al.^25^, in summary neonatal donor Keratinocytes (FSK), microvascular endothelial cells (FSEC) and fibroblasts (FSF) were isolated from foreskins (collected from new-borns approximately 5–7 days of age after routine circumcision). For experiments using adult/geriatric cells only keratinocytes were isolated from facial tissues obtained following minor dermal procedures (carried out on individuals between the ages of 70–90 years old).

Informed consent was obtained preceding collection of all skin. This was conducted under the approval of the Oxford Research Ethics Committee; reference 10/H0605/1. Following resection, the tissue was placed in transport medium (DMEM supplemented with 10% FBS, Gibco, 10500064, penicillin, streptomycin, amphotericin and gentamycin) and transported to the laboratory at ambient temperature. Neonatal and facial tissues were aseptically cut into oblongs of 5–7 mm each side in size (depending on initial tissue size) and digested overnight at 4 °C with 0.5 mg/ml Liberase DH in CnT-07 keratinocyte medium (CellnTech). Following digestion, the epidermis was peeled from the dermal layer, transferred to trypsin–EDTA, and the tissue mechanically dissociated to form a single-cell suspension. After pelleting, keratinocytes were resuspended in keratinocyte CnT-07, and seeded into petri dishes first coated with collagen/fibronectin.

From neonatal tissues, fibroblasts were isolated by placing remnants of dermal explants (de-epidermised pieces of skin) into gelatine-coated vessels supplemented with DMEM/10% FBS. FSEC’s were isolated from the neonatal biopsies by digesting the rest of the tissue in 2.5 mg/ml collagenase in HBSS (with Ca and Mg) at 37°C with frequent agitation for 1 h, followed by passing through a 70 μm cell strainer and selection using CD31 magnetic Dynabeads (Life Technologies, 11155D). Selected cells were then seeded onto a gelatine-coated flask in Endothelial Cell Growth Medium MV (PromoCell, C-22020).

### UV exposure

UV exposures were performed using a BIO-SUN UV system (Vilber Lourmat). The device has four 30 W UV-A tubes with peak output at 365 nm, and two 30 W UV-B tubes with peak output at 312 nm. Spectral irradiance was measured by a calibrated QE65000 spectrophotometer (ocean optics) coupled with a 600um optical fibre to a D7H cosine diffuser (Bentham Instruments) For the assessment of nitrate and nitrite solutions in PBS (non-cell culture-based work) a UV-B output was used, for all other experiments only UV-A emitters were used. To select the specified spectral region, the BIO-SUN output was modified by application of long pass filters (LWP) LWP345 and LWP355. Application of these filters reduced UV-B content in UV-A emission from 0.9% in unfiltered output to 0.01% and 0.05%, and UV-A1 emission below 340 nm from 17 to 1.8% and 7.6% by LWP345 and LWP355, correspondingly. The filters spectral transmittance was taken into account for dose-rate and dose estimation. A UV-A exposure dose of 9 J/cm^2^, approximately equivalent to 30 min sunlight on a UK summers day, was chosen for the experiments. It should also be noted that the BIO-SUN incorporated live dose measurement, and this was used to ensure consistent dose delivery.

### Nitrite/nitrate breakdown following UV-A/-B exposure with DAF-FM

For non-cellular based nitric oxide quantification, a non-diacetate form of DAF-FM was used. A 10 mM working solution of nitrate (NaNO_3_, 84.99 g/mol) and nitrite (NaNO_2_, 68.99 g/mol) was first prepared. In a 96-well plate, 250ul of each sample was added. DAF FM 5um (D1821, Merck Millipore) was then added to the plate, and the plates were exposed to 9 J/cm^2^ UV-A or 0.2 J/cm^2^ of UV-B. After irradiation emission of DAF-FM was read on a Biotech™ (synergy HT spectrophotometer) at 495/515 nm.

### Cyclobutane-pyrimidine dimer (CPD) ELISA

Cyclobutane pyrimidine dimers (CPD’s) are the most prevalent DNA lesion induced by UV irradiation^27^. As such, it was deemed necessary to evaluate longer UVA wavelengths in inducing this distortion to DNA. Following growth and confluence in CnT-07 keratinocyte medium (CellnTech) 1.5million FSK were seeded from stock T-75’s into 6 cm diameter plates giving 95–100% confluency after 24 h incubation. On the day of exposure, to avoid artifacts from photo-reactive compounds in the media giving rise to reactive oxygen species (ROS) from the light exposure, FSK were placed in Dulbecco’s PBS+/+ (SIGMA D8662) and exposed to UV-A, after washing first in HBSS−/−. Where filters were used, the transmittance of the filter was taken into account and dose rate adjusted to reach the same dose of 9 J/cm^2^. Immediately after irradiation cells and unexposed blanks were detached from the plates via scraping and collected in a 1.5 ml RNAse and DNAse free Eppendorf tubes. Cells were spun down at 5000×*g* for 5 min at room temperature, and the pellet was retained at − 80 °C until DNA could be extracted. DNA was extracted via use of a blood and tissue kit (Qiagen, 69504) as per manufacturers protocol. After extraction yields of DNA were measured using the Nanodrop 2000. Preliminary work suggested that a yield of around 50–200 ng/ul was optimal for the CPD ELISA assay, which involved diluting samples to 50ul with Tris–EDTA (TE) buffer. The CPD ELISA assay (Cell-biolabs, STA-322) was carried out as the protocol specified: briefly samples were placed in the heater block to 95 °C, then on ice, to convert double stranded DNA to single stranded form. The DNA standards within the assay were also simultaneously heated in the same manner to 95 °C for ten minutes then rapidly chilled on ice for 10 min. Standards and samples were added to the plate; 50ul of binding buffer was then added to wells with samples and standards. The plate covered and left overnight at room temperature on an orbital shaker. The following day after a series of washes with the wash buffer, followed by antibody stains first with primary antibody, then with secondary antibody supplied within the kit, and used at the recommended concentration. Detection reagents were then used as the protocol described with the colour change within the assay (denoting CPD’s) being monitored on a Biotech synergy HT spectrophotometer at 450 nm.

### Nitric oxide detection using DAF-FM DA

The diacetate form of DAF-FM (DAF-FM DA) is a cell permeable dye that upon entering the cell has diacetate cleaved effectively rendering the dye active. The dye can bind nitric oxide at this point and alters its fluorescence accordingly. Loading of DAF-FM DA was performed 45 min prior to nitric oxide quantification by adding 1 µl of 5 mM stock of DAF-FM DA (Molecular Probes D23844) in DMSO per 1 ml of culture media, giving a final concentration of 5 µM. After incubation the media containing DAF-FM DA was removed, and the cells washed with HBSS−/− (SIGMA, H6648) and removed from the plate by the Trypsin–EDTA solution. Cells were centrifuged and resuspended in Dulbecco’s PBS+/+ (SIGMA, D8662). For exposures 150 µl samples of the cell suspensions were then loaded into 96-well plates, with 150 µl PBS+/+ containing 50 µg/ml propidium iodide (PI) (SIGMA, P4170) added. Plates were read with a Guava EasyCyte HT flow cytometer (Merk Millipore). Cells positive for PI were excluded from the study.

### Addition of L-NAME for NOS assessment post UV-A exposure

The non-specific reversible NOS inhibitor, N (gamma)-nitro-l-arginine methyl ester (L-NAME), was selected to establish the activity of the nitric oxide synthase enzymes (NOSs) within keratinocytes after UV-A exposure. L-NAME (SIGMA, N5751), a non-specific inhibitor of NOS via arginine inhibition, was used at a concentration of 50 mM and had an incubation time of 1 h 45 min established as optimal after a series of titration experiments. Prior to addition of L-NAME cells were washed in pre-warmed Dulbecco’s PBS+/+ (SIGMA, D8662). DAF-FM DA was loaded 1 h after L-NAME addition, by adding 1 µl of 5 mM stock of DAF-FM DA (Molecular Probes D23844) in DMSO per 1 ml of culture media, giving a final concentration of 5 µM, After incubation, the media containing DAF-FM DA and L-NAME was removed, the cells washed with HBSS−/− and removed from the plate by action of the Trypsin–EDTA solution. 150 µl samples of the cell suspensions were loaded into 96-well plates, 150 µl PBS with 50 µg/ml propidium iodide (PI) (SIGMA, P4170) added, and the plates read with a Guava EasyCyte HT flow cytometer. Cells positive for PI were excluded from the study.

### RNA extraction from cells

FSKs were first grown in 6 cm diameter cell culture dishes in CnT-07 keratinocyte medium (CellnTech) to 90 or 95% confluency. Medium was removed, and the cells rinsed with Dulbecco’s PBS+/+ (SIGMA, D8662). 3 millilitres of pre-warmed PBS were then added to the cells, and the cells were exposed to 9 J/cm^2^ UV-A. Following exposure, plates were placed in fresh media and returned to the incubator. The media were changed daily, and the cells were harvested at 24, 48, 72 and 96-h timepoints. Cells were harvested via scraping into PBS+/+ followed by spinning down at 10,000 RPM removing supernatant and storing at -80° C in RNAlater (Thermo, AM7020). When all samples were collected, RNAlater was removed and a lysis buffer supplied as part of the Qiagen RNAeasy ‘mini’ kit (Qiagen, 74104) supplemented with 1% beta-mecaptoethanol was added. The sample was then lysed by spinning through a ‘QIAshredder’ column (Qiagen, 76956) at 14000×*g* for 1 min and RNA was isolated as the Qiagen RNAeasy kit protocol. Briefly, RNA was precipitated from the sample with the aid of 70% ethanol. The sample was put through a column capable of binding the precipitated RNA to its membrane. A series of washes to clean contaminants from the sample and a DNase digestion were finally performed removing residual impurities and traces of DNA that might interfere with the end QPCR results. The RNA was eluted with water, and integrity/yields assessed via the Agilent technologies 2200 Tape-station and nanodrop 2000.

### QPCR analysis of RNA for NOS isoforms

CDNA was prepared from the RNA using a high capacity CDNA kit (Applied biosystems, 4387406) as per protocol. Briefly, 10 μl of master mix was placed into a 0.5 millilitre tube. The RNA sample was also then placed into the tube, again at 10 ul, at a concentration of 100 ug/ul. The sample was centrifuged at 200 rpm for 10 s to eliminate any air bubbles and loaded into a thermal cycler. QPCR was then carried out on CDNA on the following NOS genes using the following primer sets, with HPR4 as a housekeeping gene:

NOS1 FCTGTAACCATGTCAACTATGCCA, RGTTCCAGACTCGGAAGTCGTG

NOS2 FTCATCCGCTATGCTGGCTAC, RCCCGAAACCACTCGTATTTGG

NOS3 FCAGCCATCACAGTGTTCCC, RTAGCCCGCATAGCGTATCAG

HPR4 FTCAGGCAGTATACAAAGATFGGT RAGTCTGGCTTATATCCAACACTTCG

### Western blotting

FSKs were grown in 6 cm diameter cell culture dishes in CnT-07 keratinocyte medium (CellnTech) until 90–95% confluent. Media were then removed, and the cells rinsed with HBSS−/−. Three millilitres of pre-warmed PBS were then added to the cells and cells were exposed to 9 J/cm^2^ UV-A in the BIO-SUN UV exposure system. Following exposure, plates were returned to the incubator in fresh media. Media were changed daily, and cells were harvested at 24, 48, 72 and 96 h. Cells were harvested via scraping into PBS, spinning down at 10,000 rpm, and removing supernatant. Protein was extracted from cell pellets by resuspending in 100 µl PBS and adding 100 µl 2× SDS Protein Lysis Buffer (2% SDS in 0.1 M Tris pH 8.0). The lysate was passed through a ‘QIAShredder’ Column (Qiagen, 76956), and protein concentration was measured using a Pierce BCA Protein Assay Kit (Thermo Scientific 23225) as per manufacturer’s protocol. Up to 20 µg of protein per sample lane was loaded into a 12% separating gel with a 4% stacking gel, the gel was run at 120 V for 1 h, and transferred to PVDF membrane via a dry transfer system (Trans Blot Turbo unit-Bio-Rad). The membrane was blocked with 10% milk powder in Tris-Buffered Saline-TWEEN (TBST) for 1 h at room temperature. Primary antibody for DNA damage assessment phospho-H2AX was then incubated overnight at room temperature in TBST with 10% milk powder at a dilution of 1:5000, Blots were also stained with the control antibody GAPDH (1:5000) (SANTA-CRUZ, SC25778) to ensure correct loading. Primary antibodies were detected via chemiluminescence with donkey anti-rabbit antibody (SANTA CRUZ, SC2313) at a 1:10,000 concentration following incubation for 1 h at room temperature.

### Ethics and regulatory approval

Written consent was obtained from parents or local authority representatives prior to collection of skin samples from neonates. For adult/geriatric experiments Informed consent was also obtained preceding collection of skin. This was conducted under the approval of the Oxford Research Ethics Committee; reference 10/H0605/1. All lab work carried out as part of this study was done in accordance with relevant guidelines and regulations.

## Results

### Nitric oxide production from nitrite and nitrate after UV light exposure

**Figure 1:**
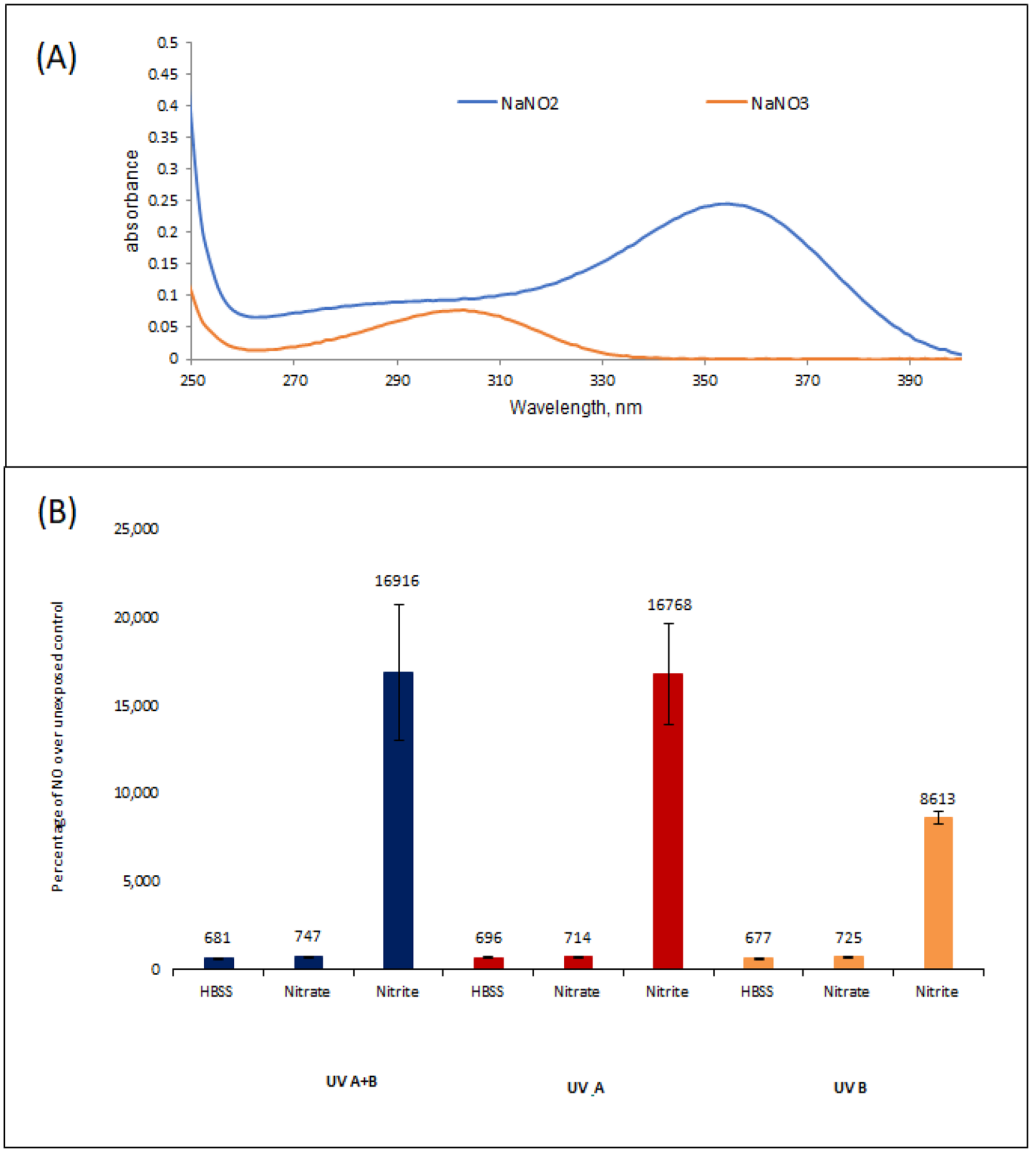
(**A**) Absorption spectra of nitrite (blue) and nitrate (orange). (**B**) Levels of nitric oxide in Hepes-buffered saline with or without 1 mmol nitrate or nitrite, following exposure to combined UV-A and UV-B (blue bars), UV-A (red bars) or UV-B only (orange bars) at 0.09 J/cm^2^ UV-B and 9 J/cm2 UV-A) levels.

Previous work has suggested that nitrate which is stored within the skin in high amounts is unaffected by long wavelength UV radiation alone. Nitrite, however, which is also present in skin but at significantly lower amounts, is susceptible to being converted to nitric oxide when exposed to UV-A^26^. Absorption spectra of these metabolites back up this notion, with only nitrite absorption being in the longer UV-A range^25,26^ (Fig. 1A). Here we show that nitrate is not broken down into nitric oxide by UV-A (315–400 nm) UV-B (280–315) or in combination (Fig. 1B) and needs other cofactors. The less stable nitrite on the other hand, is converted to nitric oxide by UV-A (315–400 nm) and UV-B (280–315 nm) (Fig. 1B). This builds on the notion that other co-factors (such as thiols, acidic conditions or nitrate reducing enzymes) are essential to aid in reduction of nitrate to nitrite allowing further breakdown to nitric oxide by UV exposure.

**Figure 2:**
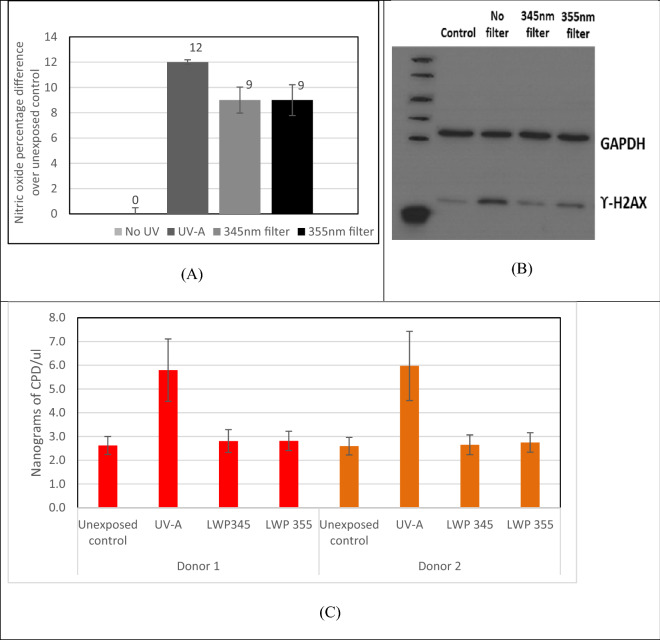
Primary neonatal foreskin keratinocytes exposed to 9 J/cm2 full spectrum UV-A, or UV-A filtered through 345 nm or 355 nm long-pass filters. All samples were compared against unexposed samples kept in the dark at the same temperature and hence subjected to minimal/low levels of background exposure as possible. (**A**) Nitric oxide produced following exposure; (**B**) DNA damage via western blotting of gamma H2AX; (**C**) CPD levels elicited by exposure: donor 1 (red bars), donor 2 (orange bars).

While there are potential benefits of generating nitric oxide by UV-A, this has to be balanced by the reported potential DNA damaging action of UV-A exposure. To further characterise and define the wavelength range that impinge on these two distinct outcomes (nitric oxide production and DNA damage), the UV-A spectrum was fractionated using long-pass filters with cut-offs at 345 nm and 355 nm. Filtered UV-A with wavelengths near the peak of the nitrite absorption spectrum (340–360 nm) induced a similar level of nitric oxide production in keratinocytes as that by the whole UV-A spectrum (Fig. 2). Measurement of DNA damage via phospho-H2AX (Fig. 2B) and cyclobutane pyrimidine Dimers (CPD) (Fig. 2C) demonstrates that application of longpass filters reduced DNA damage to levels comparible with the unexposed control cells. These experiments clearly show that the filtered UV-A spectrum activates NO production without eliciting measurable DNA damage.

### UV-A induces delayed second wave (dark reaction) of nitric oxide production

**Figure 3:**
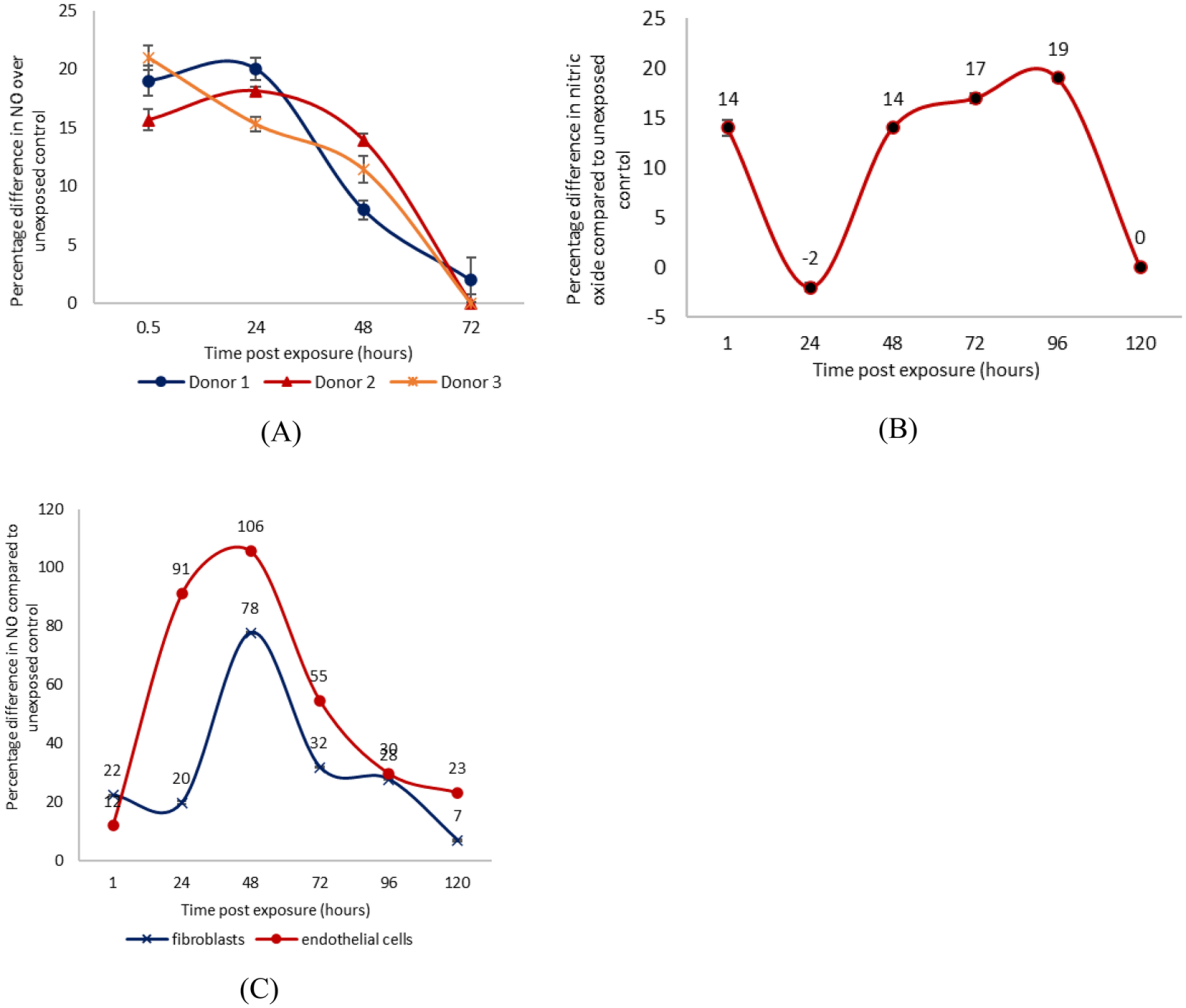
Increase of nitric oxide in primary human skin cells due to exposure 9 J/cm^2^ of UV-A compared with unexposed control cells at various time points after exposure. (**A**) Keratinocytes derived from three independent neonatal donors. (**B**) Keratinocytes derived from skin of adult donor. (**C**) Fibroblasts and endothelial cells.

Although the release of NO in response to sunlight exposure can be beneficial, the extent of this benefit may be transient and limited if this occurs only during the period of exposure. In order to understand the dynamics of UV-A-induced NO production, timepoints of 1, 24, 48, 72 and 96 h post-irradiation were chosen to monitor nitric oxide production in these cells, which were subsequently cultured in the dark. To ensure reproducibility the experiments were performed at least three times in triplicate with keratinocytes from three different donors. Post-exposed cells at each timepoint were compared against unexposed control cells at the corresponding timepoint. As with the previous experiment described above, a cell permeable DAF-FM diacetate was loaded into cells for 45 min at each timepoint prior to analyses. Florescence from DAF-FM bound to NO was then monitored via flow cytometry and dead cells were identified with propidium iodide which allowed the gating and exclusion by FACS. Nitric oxide produced by keratinocytes was observed for a prolonged period of time after the initial exposure to UV-A radiation (Fig. 3A) and the rapid rise in NO during exposure (0.5 h) lasted for at least a day before gradually declining in the next few days down to the pre-exposure levels. Although the NO production profile of the three donor keratinocytes are not identical, as is expected when experimenting with primary cells from different donors, they all clearly revealed a similar trend which is unexpected extension of NO production in the dark after UV exposure peaking between 30 min and 24 h after exposure, and lasting for 72 h.

To ascertain whether this effect was unique to the age or site of the skin from which the keratinocytes were isolated, which in this case were all derived from neonatal foreskins, keratinocytes isolated from the facial skin of an adult donor were exposed to 9 J/cm^2^ UV-A and nitric oxide generation was measured up to 5 days after exposure. Prolonged elevation in NO production was again observed, despite the noticeable difference of a curious drop in the 24 h timepoint, and a prolonged level of upregulation lasting after the 72-h period. It suggests the phenomenon seen in neonatal foreskin cells is not unique to this type of keratinocyte or location of skin sample and is likely a feature of keratinocyte in general regardless of age or provenance (Fig. 3B).

Experiments were repeated with microvascular endothelial cells and fibroblasts, which are the major constituent cells of the skin after keratinocytes and were isolated from neonatal foreskin donors. Again, a late rise in NO production in the dark was seen days after exposure to UV-A, the peak being higher and later than that seen in keratinocytes. Demonstrating that the long-term nitric oxide production witnessed in keratinocytes is not unique to this cell type (Fig. 3C).

### Dark reaction generation of NO is accompanied by increased expression of multiple NOS isoforms and mediated by their activities

**Figure 4:**
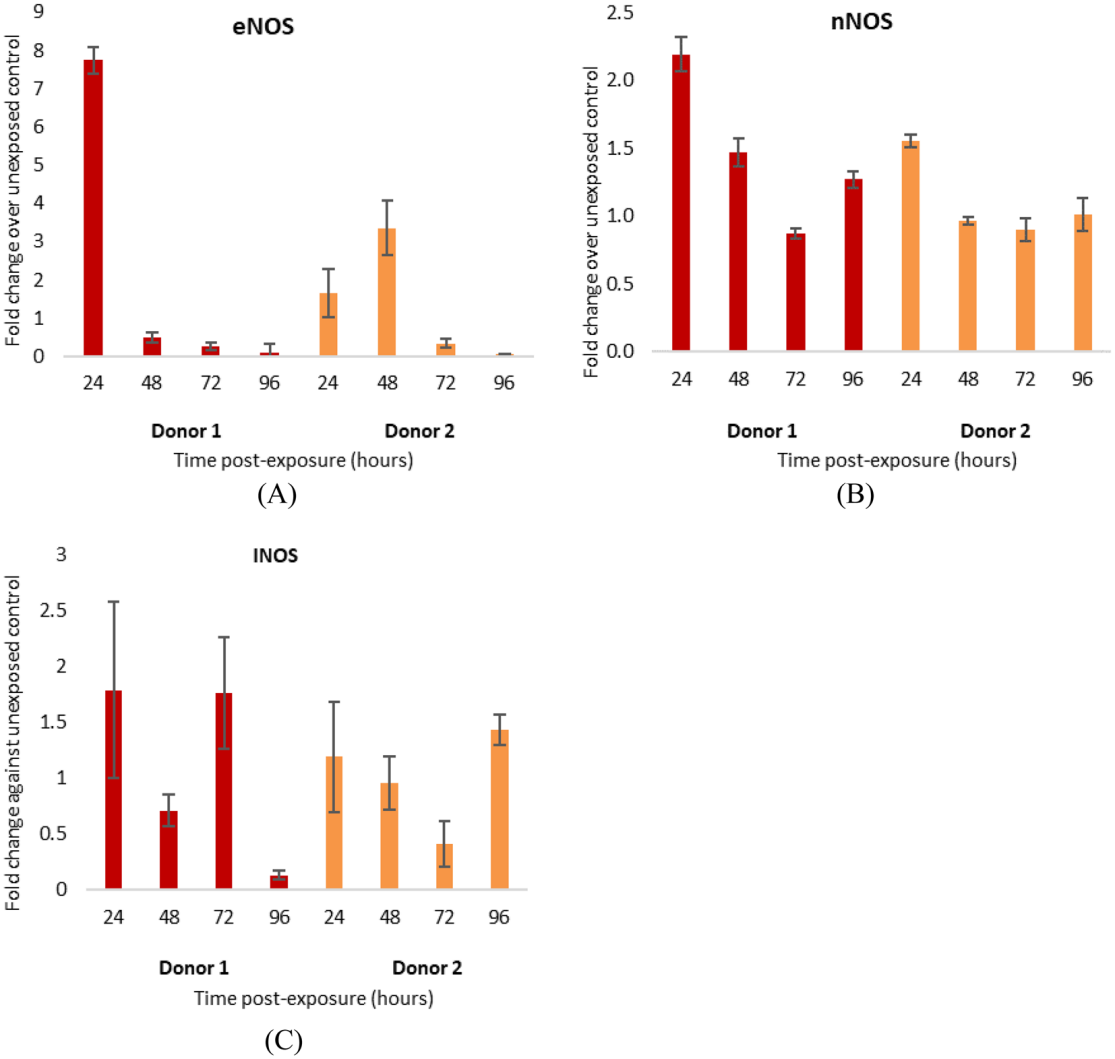
Fold change in eNOS (**A**), nNOS (**B**) and INOS (**C**) gene expression in primary human keratinocytes, up to 96 h after UV-A irradiation. Keratinocyte donor 1 in red and keratinocyte donor 2 in yellow.

The observation that NO production increased long after exposure to UV-A is unexpected. The robustness of this observation is not in doubt given that it was consistently observed in three different skin cell types of different ages, from different parts of the anatomy and from multiple different donors. To investigate whether this prolonged NO production in the dark post exposure, could be mediated by enzymes we measured their expression using QPCR with primers for eNOS, nNOS and iNOS. The results show that the expression of the genes encoding these three NOS isoforms were significantly upregulated at early timepoints 24–48 h after irradiation (Fig. 4A,B). Surprisingly, iNOS, commonly associated with the late stage inflammatory effects of UV-B exposure, showed a non-uniform level of upregulation in both donors (Fig. 4C).

**Figure 5:**
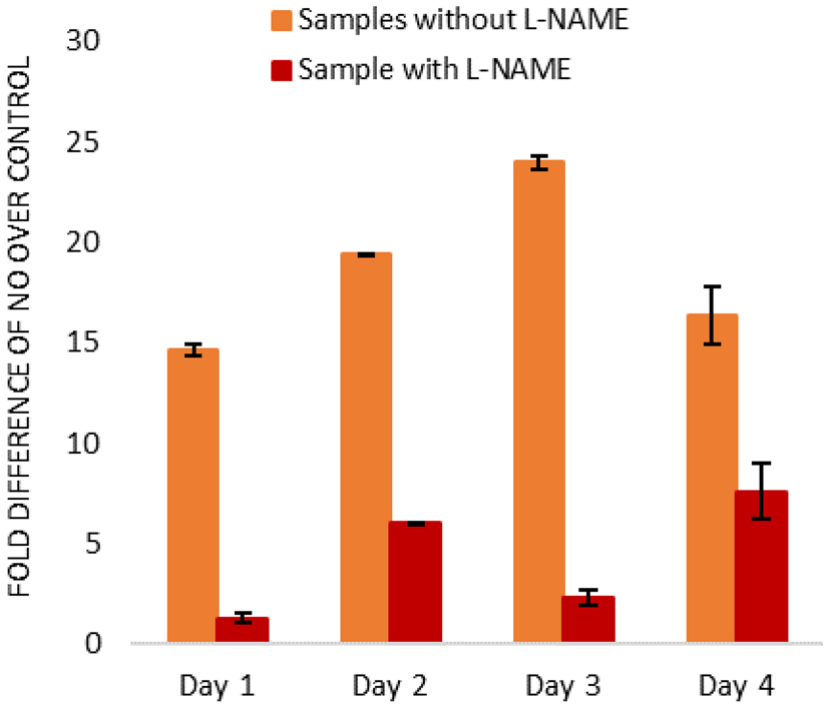
Increased NO production at various times after exposure of primary human keratinocytes to UV-A.

This suggests that upregulation of nitric oxide post-UV-A exposure is not solely due to one NOS isoform, but the combined effect of all isoforms expressed in cells. The erratic nature of the increased expression of these enzymes does not allow a definitive conclusion as to which one is dominant and requires further investigation. To test whether augmented NOS were responsible for the prolonged production of NO, we added N (gamma)-nitro-L-arginine methyl ester (L-NAME) which inhibits all NOSs to UV-A exposed cells. At set timepoints over a 5-day period, with the use of DAF-FM diacetate to quantify NO, the contribution of the NOS enzymes in the production of nitric oxide could be monitored. Although the upregulation of NO from exposed samples without L-NAME differed in magnitude from the data highlighted in Fig. 2 (with a longer upregulation lasting greater than 72 h) a biphasic response was still witnessed, and addition of L-NAME at a concentration of 50 mM 1 h prior to DAF addition resulted in a significant reduction of nitric oxide at all timepoints between 24 and 120 h post irradiation (Fig. 5). This suggests that unlike the initial rise in nitric oxide that is metabolite-based, NOS activity is responsible for the rise in nitric oxide during the dark reaction. There is nevertheless residual NO production in the presence of L-NAME, suggesting the other non-NOS pathways might also be involved in the dark reaction in generation of NO.

## Discussion

A study by Dejam et al., assessing the ability of UV-A to breakdown nitrogen metabolites within the skin, suggests that nitrate remains untouched by UV irradiation alone, requiring the presence of co-factors such as thiols to derive nitrite from nitrate prior to NO release^28^. Other studies back up this notion, highlighting that thiols, pH changes and enzymes such as nitrate reductase are essential cofactors needed prior to light exposure for nitrate breakdown^29,30^. Conversely metabolites such as nitrite and nitroso-thiols stored within the epidermis at much lower levels are the substrates broken down by UV-A alone to produce NO^28,31^. Recent work by Paunel et al. further builds on this suggestion, highlighting that these metabolites have a different decomposition rate for nitric oxide release with inhibitory compounds showing nitrite and nitroso-thiols react differently to UV-A, with nitrite providing a longer lower level of NO release following decomposition and nitroso-thiols providing a more rapid, pronounced elevation of nitric oxide after UV-A exposure^30,31^.

Our own experiments in this area, assessing the breakdown of nitrate and nitrite in PBS after UV-A or -B exposure support these claims, with results suggesting that nitrate is stable when exposed to UV-A and UV-B irradiation alone, with nitrite deriving the bulk of NO. This is interesting as nitrate is stored at significantly higher levels (100 μM) than nitrite (15 μM) and s-nitroso thiols (7 μM) within the skin^30^. Hence the vast proportion of nitrogen-based metabolites stored within the skin are not directly photo-liable^28^, instead requiring cofactors such as nitrate reductase enzymes to aid in conversion to a photo-active form^31,33^. This dependence of epidermal nitrate on other factors to become photo-liable is consistent with the fact that NO at high concentrations is harmful in vivo^37^.

We demonstrate here that UV-A induced NO upregulation is generated through the metabolite-dependent pathway, as removal of shorter wavelengths (280–340 nm) previously reported to induce NOS activity^34^ invoked little change in NO production compared to exposure to full spectrum UV-A.

Research by Cuspundi et al. and others suggests seasonal variations in blood pressure are more pronounced as age progresses and in demographics with metabolic diseases such as diabetes^35–37^. Alongside this notion, it is understood that the action of the NOS enzyme is increasingly dysfunctional in these demographics, resulting in decreased production of nitric oxide, with concomitant increased production of the toxic by-products, superoxide (O-) and peroxynitrite (ONOO)^2,13^. Enzyme ‘uncoupling’ is understood to be responsible for these effects, through NOS changing conformation from a dimeric to a monomeric form^2,13^. Mouse studies back up claims that nitrite within the skin can compensate for this loss of NOS activity^37^. As such it is hypothesized, (albeit yet to be tested), that exposure to UV-A could feedback into the enzyme-based pathway via these mechanisms instigating vasodilation through liberation of NO and downregulation of ROS from NOS. Experiments showed that levels of DNA strand breaks (ϒ-H2AX) and pyrimidine dimers were not augmented by long wavelength UV-A and supported previous work by Holliman et al. and others^25,38,39^ who showed that shorter UV wavelengths were at least 10,000 times more photodamaging to DNA than UV-A, causing at least 250 times more double-stranded DNA breaks and 15,000 times more CPD’s than UV-A alone^25^. This may also be of huge significance in a clinical setting whereby current technology developments may allow tailoring of simulated light allowing nitric oxide release without negative effects associated with shorter wavelength UV. In addition to this, the elderly, diabetics, those with reduced liver function or other groups with metabolic problems potentially associated with diminished nitric oxide function may also benefit from exposure of the skin to radiation in this spectral range (^38,40,41^). The available published data on the persistence of nitric oxide production after light exposure appear to be divergent. This may be due to the fact that studies utilise dissimilar light sources with different spectra, doses and dose rates^30,42^. In addition, the involvement of largely young and fit individuals within in vivo studies, and not individuals with altered enzymatic NOS production, possibly means that effects derived from the metabolic pathway may be negligible in this age group. More recent studies in which a dose equivalent to 30 min Mediterranean sunlight was used seem to broadly infer that UV-A irradiation gives a transient 30–60-min burst of NO release post exposure^25,42,43^. In comparison, our in vitro work, which unlike these earlier studies also assessed later timepoints of NO production post UV-A exposure, suggests that UV-A mediated NO release from the skin is not a ‘transient’ effect, but carries on for many hours after exposure. This mechanism was also demonstrated for other skin cells situated below the epidermis: endothelial cells and fibroblasts also increasing NO production many hours to days after exposure. This falls in line with previous data reported by Holliman et al. suggesting all skin cells are capable to some extent of activating a nitric oxide producing pathway after UV-A exposure^25^. The continued release of NO in the dark was somewhat surprising as the primary metabolites, nitrite and nitroso-thiols, have relatively short half-life in vivo, hence prolonged breakdown of these metabolites for a considerable length of time after exposure is implausible. As such, our focus turned to the enzymatic NOS pathway known to be associated with this phenomenon. It is likely that this pathway may, in fact, instigate this profound effect as others have found NO to be significantly upregulated via NOS at least 24 h following UV-B exposure^44,45^.

Work with L-N omega-Nitro-l-arginine methyl ester hydrochloride (L-NAME), a cell permeable arginine analog capable of blocking the NOS enzymes, confirmed our hypothesis. The results here suggested that the late rise in NO was in fact enzyme-dependent; but unlike UV-B-mediated studies that attribute iNOS as the major mediatory factor in the rise of NO^33,44,45^, UV-A appeared to upregulate eNOS and nNOS in a similar manner 24–48 h after exposure. iNOS’s role following UV-B exposure could, in part, be due to the much more damaging effect of this wavelength range eliciting increased production of pro-inflammatory cytokines when compared with exposure to UV-A^44^.

Although unexpected, this is not the first time that calcium-based NOS (cNOS) isoforms eNOS and nNOS, nitric oxide and UV-A irradiation have been linked to potential vasodilatory effects. Research implies heat shock proteins (HSP) such as HSP-32 and HSP-90 can be upregulated by UV-A exposure. This is of significance as these, in turn, have been shown to readily induce positive phosphorylation of cNOS at ser177, effectively aiding in giving rise to increased nitric oxide^46–49^. Additionally, UV-A has also been suggested to alter calcium levels in cells. As cNOS (through calmodulin domains) requires upregulation of calcium in order to become active, this may also aid in nitric oxide production from eNOS and nNOS^47^. It is therefore possible that the UV-A induced dark reaction could control cNOS homeostasis in a number of ways, with interplay between these and other UV-A induced cNOS mechanisms upregulating NO long after exposure. These questions require further investigation; like most highly effective enzymes, NOS is expressed at very low levels and undergoes additional post-translational processes such as dimerization and phosphorylation^2–4^, hence there is still a lot to uncover in relation to how this enzyme functions in vivo.

Finally, as reports suggest cardiovascular disease to be a disease of the advancing age, with incidence of heart failure doubling with each decade of life^50^, it was essential to compare expression of nitric oxide from initial data (utilising neonatal keratinocytes) against aged donors (utilising geriatric keratinocytes). This was important, if the NOS enzymatic pathway was involved in this late stage upregulation, as in geriatric skin cells the presence of monomeric NOS (through loss of its cofactor tetrahydrobiopterin) has been categorically shown to elicit a decline in NO and in turn increase oxidative stress, through increased superoxide and peroxynitrite production^37,49,50^. As such, an upregulation in NOS in aged individuals may, in comparison to the metabolite driven pathway, promote release of toxic superoxide and peroxynitrite, and not NO at later timepoints. Results generated from UV irradiated geriatric donors suggested a prolonged production of nitric oxide did occur post-exposure lasting for several days. If the specificity of dyes used for this work holds true, and only nitric oxide has been bound here (and not analogs such as peroxynitrite), these results suggest that the prolonged effects of UV-A exposure may be vital as we age, permitting positive vasodilatory effects from sunlight to occur. Further work is necessary to evaluate this hypothesis, adding further credence to epidemiological data suggesting the sunlight hours inversely correlate to cardiovascular disease status globally.

## Conclusions

The duration of nitric oxide release in skin cells after UV-A exposure is not well studied, with emphasis instead on UV-B mediated induction of NO at later stages predominantly through inflammatory mediators upregulating iNOS. The current study demonstrated persistence of nitric oxide release after moderate UV-A exposure, peaking around 48 h post exposure; this effect was shown in keratinocytes, fibroblasts and endothelial cells of both neonatal and aged donors. It confirmed the hypothesis that UV-A exposure appeared to upregulate cNOS alongside iNOS. Release of nitric oxide in the skin cells induced by a moderate exposure to UV-A in sunlight may be especially beneficial for some demographic groups such as the elderly, hypertensive patients or those with impaired nitric oxide function, not only during exposure but many hours and days after that (Supplementary information S1).

## Supplementary Information

Supplementary Information.
