# Supplementary material for: Post-exposure persistence of nitric oxide upregulation in skin cells irradiated by UV-A
Source: Sci Rep. 2022 Jun 8;12:9465. doi: 10.1038/s41598-022-13399-4 (PMC9177615; doi:10.1038/s41598-022-13399-4)
Supplement: Supplementary file 1 — Supplementary Information. [file 41598_2022_13399_MOESM1_ESM.docx]

**Post-exposure persistence of nitric oxide upregulation in skin cells irradiated by UV-A**

Gareth Hazell^1^, Marina Khazova^1^, Howard Cohen^2^, Sarah Felton^3^, Ken Raj^1^

*UK Health Security Agency, Chilton, Didcot, OX11 0RQ, UK*

*^2^Elizabeth House, 515 Limpsfield Road, Warlingham, Surrey CR6 9LF, UK*

*^3^Oxford University Hospitals NHS Foundation Trust, Old Road, Oxford OX3 7LJ, UK*

** Corresponding author:*

*gareth.hazell@ukhsa.gov.uk ,*

*UK Health Security Agency,*

*Chilton,*

*Didcot,*

*OX11 0RQ,*

*tel +44 (0)1235 825 132*

Short title: UV-A and prolonged upregulation of NO in human skin cells.

**Supplementary figure 1.**

Complete scan of western blot membranes that were cropped for inclusion in figure 2b

| Lane | Sample |
| --- | --- |
| 1 | Ladder |
| 2 | Unexposed keratinocytes |
| 3 | Keratinocytes exposed to whole spectrum UVA |
| 4 | Keratinocytes exposed to UVA with LWP345 |
| 5 | Keratinocytes exposed to UVA with LWP355 |
| 6-9 | Samples unrelated to experiment – different project |


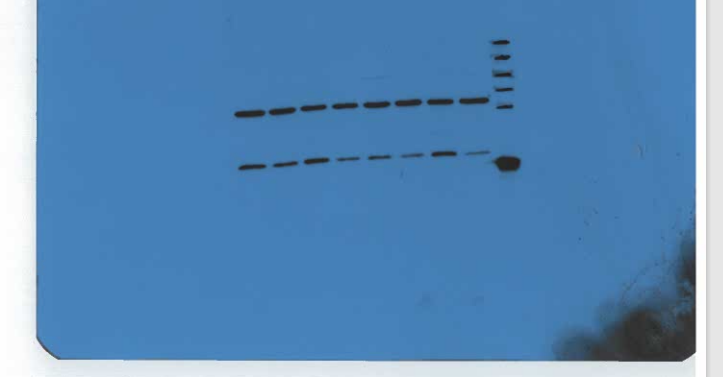


Samples from an unrelated experiment

GAPDH

Gamma H2AX

10 kda

20kda

37kda

50 kda

100 kda

250 kda

Layout of gel:

**Supplementary figure 2**

Spectral irradiance of filters used for work, alongside output of the BIOSUN.
